# Photosensitive disorders in HIV

**DOI:** 10.4102/sajhivmed.v18i1.676

**Published:** 2017-08-31

**Authors:** Karen Koch

**Affiliations:** 1 Wits University Donald Gordon Medical Centre, Johannesburg, South Africa

## Abstract

Photosensitive disorders are common, affecting up to 5% of HIV-positive patients. HIV itself induces photosensitivity but photoaggravated drug reactions, porphyria cutanea tarda and nutritional disorders such as pellagra are also more common in patients with HIV. In South Africa, actinic lichenoid leukomelanoderma of HIV is a unique photosensitive disorder which is associated with advanced HIV. It is important to be able to recognise these conditions and withdraw photosensitising medications wherever possible.

## Introduction

HIV is known to cause photosensitivity, and approximately 5% of patients with HIV have some form of photosensitive dermatitis.^1^ These conditions include photosensitive drug reactions, chronic actinic dermatitis (CAD), pellagra, lichenoid photoeruptions, porphyria cutanea tarda (PCT), pseudoporphyria, photoaggravated granuloma annulare and actinic prurigo.^2^ Actinic lichenoid leukomelanoderma of HIV – a photosensitive dermatological condition specific to South Africa – represents a possibly unique entity seen in our clinical setting.^3,4^

## What is photosensitivity?

**FIGURE 1:**
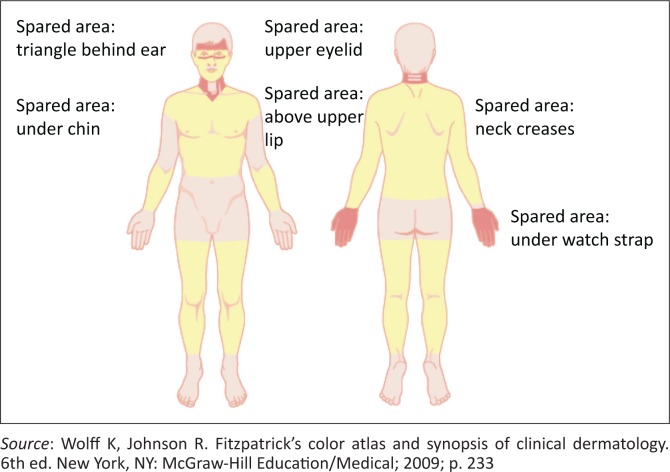
Photosensitive distribution.

Photosensitivity is an inflammatory reaction caused by an abnormal response to ionising radiation. Photosensitive rashes (as depicted in Figure 1)^5^ affect sun-exposed areas and are more pronounced on the face, ears, scalp, posterior neck, upper back, the ‘V’ area of the chest, extensor surface of arms and dorsal hands. The key in identifying photosensitivity is involvement of sun-exposed areas with sparing on the face of the nasolabial folds, eyelids and submental areas. Some skin disorders are primarily related to ionising radiation, whilst others are simply aggravated by sun exposure.^5^

## Pathogenesis

The pathogenesis of photosensitivity in HIV is multifactorial. Ultraviolet (UV) radiation causes the production of reactive oxygen species in the skin, resulting in DNA damage and cell destruction. Oxygen-free radical scavenging pathways are dysfunctional in HIV because of a host of factors, including a relative glutathione and thioredoxin deficiency, alterations in the dietary absorption of elements such as vitamins and flavonoids, and liver damage secondary to medications.^2^ Certain medications such as trimethoprim-sulphamethoxazole, non-steroidal anti-inflammatory drugs (NSAIDs) and antiretrovirals can be photosensitising and may increase the risk of photodermatitis.^1,6^

Photosensitivity typically occurs in patients with a CD4 T-lymphocyte count less than 200 cells/µL and often below 50 cells/µL^7^ and is 6.68 times more likely to occur in African Americans.^1^

## Photosensitive drug reactions

Photosensitive drug reactions can be either photoallergic or phototoxic, both of which occur more frequently in the setting of HIV. Both drug reactions can result from oral or topical medications. Prescribed, over-the-counter medications and herbal supplements may all be inciting agents.^8^

A phototoxic reaction is a non-immunologic reaction that looks like exaggerated sunburn, whilst a photoallergic drug reaction is an eczematous immune-mediated reaction to medications.^8^

**FIGURE 2:**
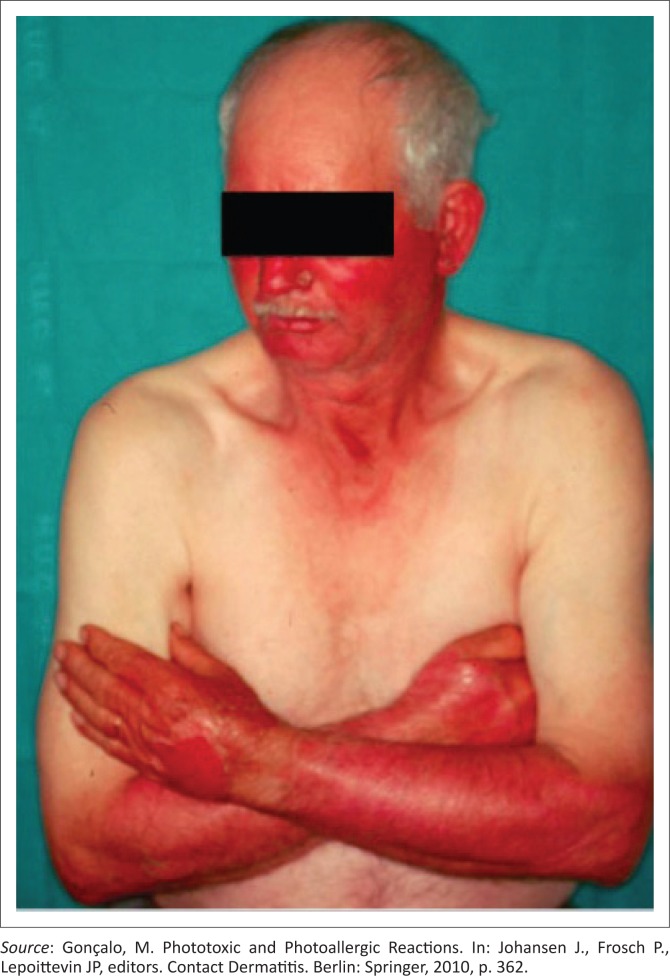
Phototoxic drug reaction.

Phototoxic drug reactions (Figure 2) occur because of a variety of medications. In the setting of HIV, sulphonamides (sulphamethoxazole and trimethoprim) and NSAIDs are the most likely causative agents. Phototoxic reactions usually occur within minutes to hours of exposure. The rash causes a burning sensation rather than pruritus. The rash is typically clearly limited to areas of sun exposure.^9^

Photoallergic drug reactions are mediated by type IV delayed hypersensitivity and occur 24 h – 48 h after UV exposure. In the setting of HIV, they are most likely to be caused by sulphonamides, NSAIDs or pyridoxine.

Efavirenz has also been reported to cause photoallergic dermatitis.^10^ Clinically, the lesions occur on sun-exposed sites but are not as well demarcated as phototoxic reactions. They are also eczematous and pruritic.^9^

Treatment involves identifying and removing the causative agent, strict sun protection, use of potent topical corticosteroids and symptomatic relief. When selecting an antihistamine, it is important to avoid using older generation medications such as promethazine as these can cause or aggravate a phototoxic or a photoallergic drug reaction.^11^

## Chronic actinic dermatitis

**FIGURE 3:**
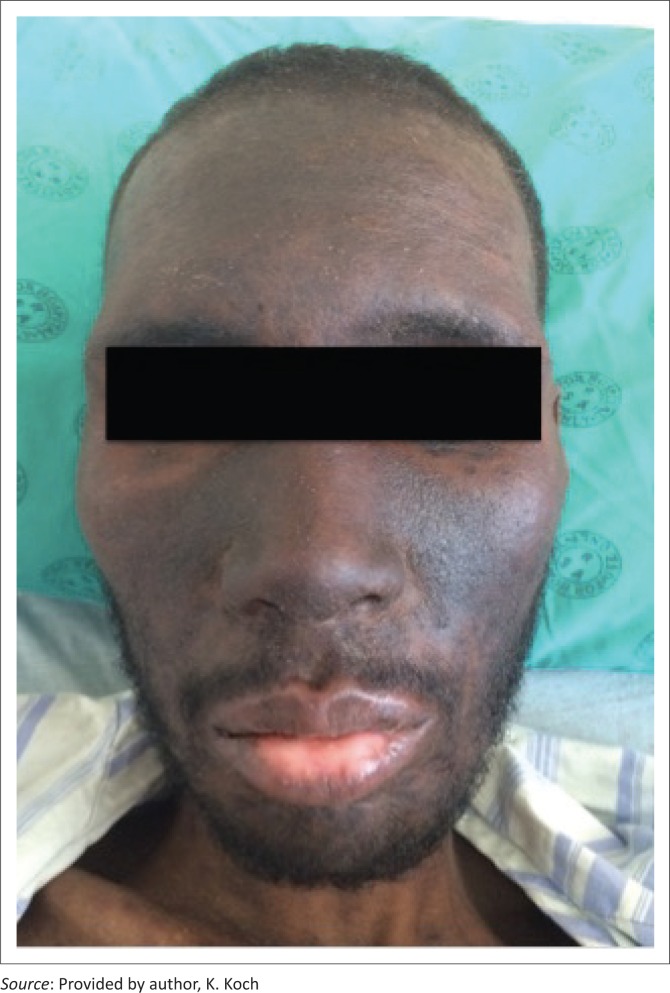
Chronic actinic dermatitis.

CAD encompasses a spectrum of photodistributed conditions including photosensitive eczema, actinic reticuloid photosensitive eczema and persistent light reaction.^8^ Some authors believe it to be the end stage of ongoing photosensitivity or persistent light reactivity in the absence of any known photosensitisers. It may occur in association with ultraviolet A (UVA) or ultraviolet B (UVB) exposure (Figure 3).^12^

It usually presents as thickened scaling patches and plaques, often becoming confluent to involve large areas of the body. The condition is common in men over the age of 50 and with dark complexion. It may progress to involve unexposed sites on the body, resulting in erythroderma. It often heals with marked depigmentation resembling vitiligo. It may be an early sign of HIV.^2,8^

Histologically, a spongiotic reaction pattern is often seen.^13^ Photosensitising agents need to be excluded in order to make the diagnosis. Treatment is difficult. Strict sun avoidance, barrier-type sunscreens containing zinc or titanium, topical corticosteroids and oral H1-type antihistamines all form part of the treatment.^12^

## Actinic lichenoid leukomelanoderma of HIV

**FIGURE 4:**
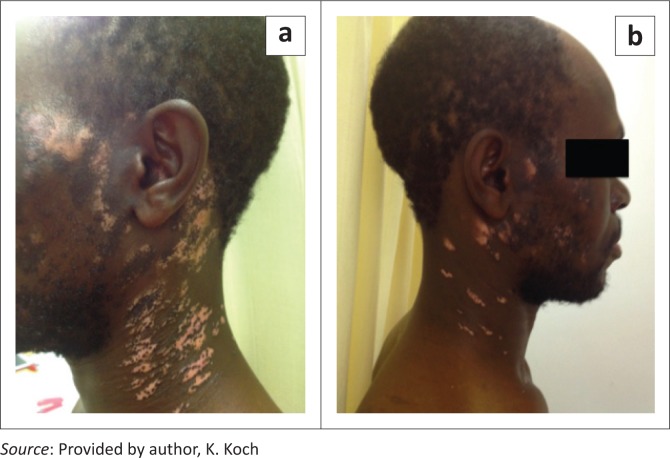
Actinic lichenoid leukomelanoderma (a and b) of HIV.

Actinic lichenoid leukomelanoderma (Figure 4) of HIV has been described in both Johannesburg and Bloemfontein.^3,4^ Unfortunately, both case series have not been published. The condition presents in the setting of advanced HIV and in the absence of exposure to any photosensitising medications.

The skin lesions resemble discoid lupus erythematosus, with well-circumscribed scaly plaques arranged symmetrically in photodistributed areas. The plaques normally start as violaceous hyperpigmented macules which progress to have central vitiligo-like depigmentation or erythematous discolouration with peripheral hyperpigmentation. The central area of the plaques may be quite thickened.^3,4^

Histologically, the skin lesions differ from lupus with lichenoid changes, including irregular acanthosis, saw-toothing of rete ridges, an interface dermatitis with necrotic keratinocytes, pigment incontinence and dermal inflammation.^3,4^

The condition is associated with low CD4 counts, and treatment with antiretroviral therapy will theoretically result in improvements. Van Rensburg and Sinclair reported a good response to topical corticosteroids and oral chloroquine at a dose of 200 mg daily.^4^ Strict broad-spectrum photoprotection is essential, as it remains unclear whether UVA, UVB or both are responsible for triggering this condition. In patients already on antiretrovirals, it is important to check for drug compliance or viral resistance.

## Lichenoid photoeruptions

**FIGURE 5:**
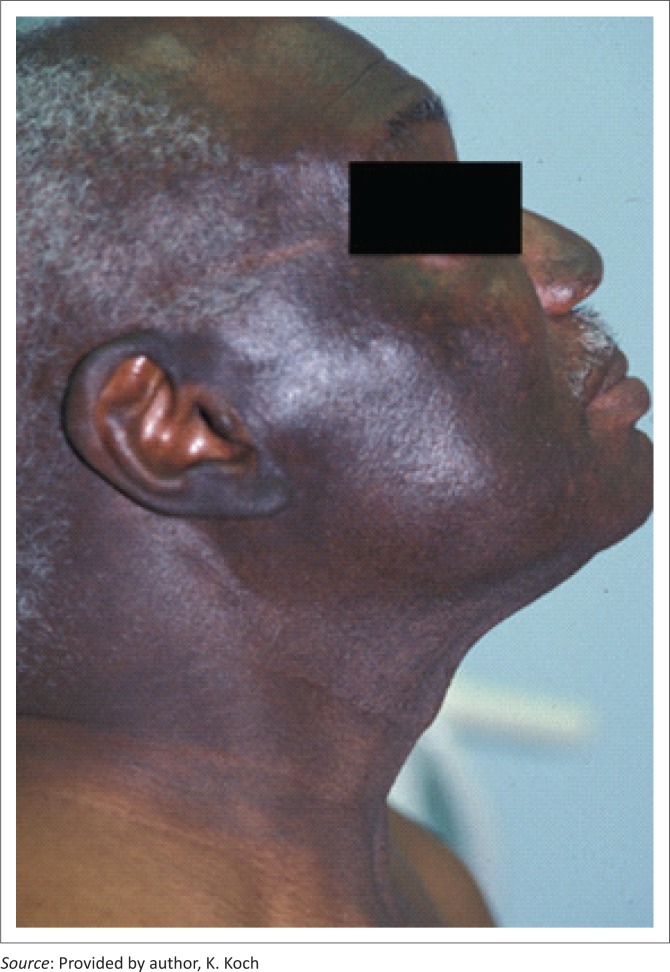
Lichenoid photoeruption.

Lichenoid photoeruptions (Figure 5) present as violaceous, flat-topped papules and plaques that resemble lichen planus. It occurs on the face, dorsum of the forearms, hands and the lower lip. It spares the mucosa and this can be helpful in distinguishing it from lichen planus.^14^

Lichenoid photoeruptions may be idiopathic but have been linked to the use of NSAIDs or sulphamethoxazole and trimethoprim. Histologically, a lichenoid pattern aids in the diagnosis.^14^

The skin lesions most often progress from red to slatey grey colour. The final dark pigmentation of the skin tends to persist for several months even after withdrawal of the offending agent. Strict sun avoidance and sunscreen are important in recovery. Patients need to be advised of lengthy recovery process.^7^

## Pellagra

**FIGURE 6:**
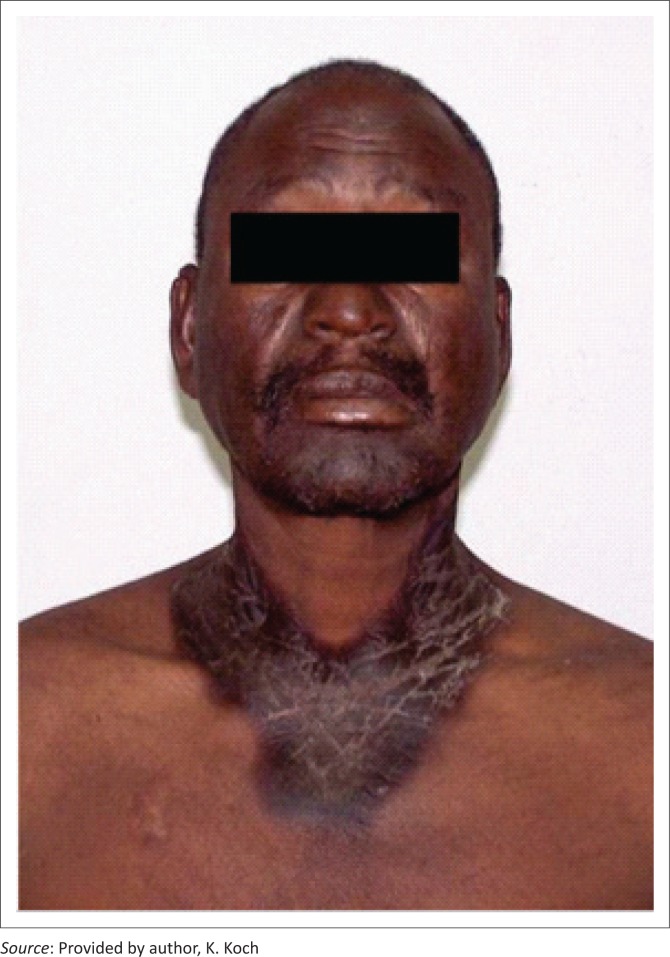
Pellagra.

Pellagra (Figure 6) is caused by a deficiency of niacin (also known as vitamin B3) or its active metabolites. HIV infection alone as well as isoniazid and pyrazinamide treatment have both been shown to cause pellagra-like disease.^15^

Niacin is produced in the body from the amino acid tryptophan. In HIV, oxidation of tryptophan along the kynurenine pathway, because of chronic inflammation, is considered to be the main cause of tryptophan depletion and hence niacin deficiency. In addition, malnutrition as a result of malabsorption and chronic diarrhoea in patients with HIV may also contribute to the disease. In South Africa, tryptophan deficiency has been associated with lower CD4 counts and patients not yet on ART.^16^

Niacin is essential for numerous processes including glycolysis, amino acid metabolism and the formation of high-energy phosphate bonds. This is why niacin deficiency tends to affect areas of rapid cell turnover including the skin and the gastrointestinal system. The classic triad of pellagra is diarrhoea, dermatitis and dementia.^17^

In the initial phase, pellagra may resemble severe sunburn with erythema and blistering in sun-exposed areas. These skin lesions resolve leaving red–brown discolouration. Typically, the patient has symmetrical superficial scaling and pigmentary discolouration on the face, neck and forearms. The characteristic discolouration over the neck and upper chest is also known as Casal’s necklace.^8^

Left untreated, hallucinations, psychosis, seizures, dementia, neurologic degeneration and coma may develop. The disease may be fatal.^8^ There are currently no tests or laboratory markers to diagnose pellagra. A high index of suspicion is required to make the diagnosis.^17^

Niacin supplementation as well as the use of antiretrovirals has been shown to reverse HIV-associated niacin deficiency.^18^ Nicotinamide at a dose of 100 mg three to four times daily should be used until clinical symptoms have completely resolved.^17^

## Porphyria cutanea tarda

**FIGURE 7:**
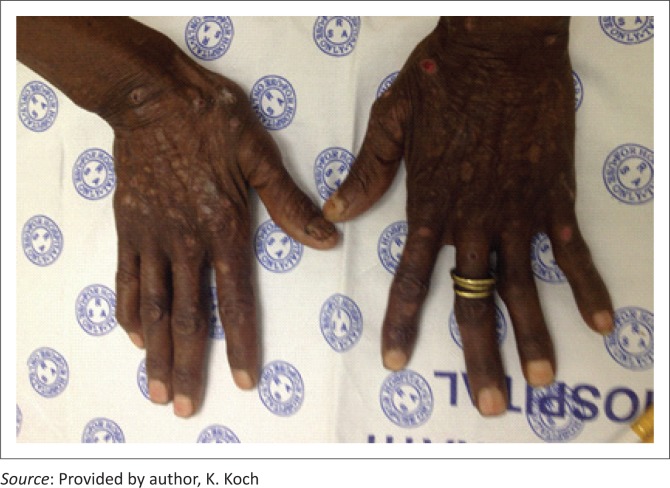
Porphyria cutanea tarda.

PCT is the commonest of the porphyrias and is because of reduced enzymatic activity of uroporphyrinogen decarboxylase (Figure 7).^7^

Accumulation of photosensitising uroporphyrins within the skin causes a blistering rash with secondary scarring on sun-exposed sites, particularly the dorsum of the hands. Other symptoms include pruritus, increased skin fragility, hypertrichosis and sclerodermoid changes.^19^

The mechanism of PCT in HIV is thought to be HIV-induced changes in porphyrin metabolism and liver injury in the setting of co-infection with hepatitis C.^20^

The diagnosis of PCT is largely aided by a skin punch biopsy for histology and direct immunofluorescence. However, measuring actual porphyrin levels in the blood, urine or stool is critical to make an accurate assessment.^21^

The differential diagnosis of PCT is pseudoporphyria, a phototoxic drug reaction which clinically and histologically mimics PCT. Pseudoporphyria occurs most commonly because of NSAIDs and is more common in patients on dialysis or infected with HIV.^11^

Patients should be managed in conjunction with a haematologist. Any medications potentially causing liver injury should be avoided as should any alcohol consumption. Underlying hepatitis must be sought and treated. Treatment consists of twice weekly low-dose chloroquine (125 mg per week) or phlebotomy to reach a target haemoglobin of 10g/dL. Strict sun avoidance is required. Patients with PCT react to visible light rather than UV, making photoprotection particularly challenging. Physical barrier sunscreens containing titanium dioxide or zinc oxide are preferable.^21^

## Management of photosensitive dermatoses

It is important to exclude drug-related causes in any form of photosensitive skin condition. An antinuclear antibody (ANA) test should be done to exclude cutaneous or systemic lupus erythematosus. A skin punch biopsy and immunofluorescence can be helpful in establishing a diagnosis. Photo-patch testing should be used if a photoallergic drug reaction is suspected.

Photoprotection, sun avoidance and strict sunscreen use are essential in all forms of photoaggravated skin conditions. Sunscreens should be both UVB and UVA protective. The labelling of sunscreens can be confusing. ‘Broad spectrum’ does not necessarily mean UVA cover. A higher sun protection factor (SPF) indicates the degree of UVB cover and not the degree of UVA protection. Sunscreens which contain physical photoprotective agents like titanium or zinc are preferable.

Directed therapies will depend on the exact nature of the skin condition. Use of a mild cleanser and emollients can help soothe the skin. Topical corticosteroids are useful during the acute phase of most photosensitive skin conditions. Antihistamines are important in relieving pruritus but second-generation agents are safer.

Patients should be advised that post-inflammatory hyperpigmentation resulting from any photosensitive condition may take many months to entirely resolve.

## Ethical considerations

The patients participated by providing their signed consent.

## Conclusion

Photosensitive disorders are common in patients with HIV affecting as many as 5% of all people. Photosensitivity can cause significant distress, often healing with disfiguring hyperpigmentation. Photosensitivity can herald a more serious underlying issue such as porphyria cutanea tarda or pellagra. Early recognition of a photosensitive drug reaction can allow for drug withdrawal and early treatment, alleviating the degree of skin damage.
